# Accuracy of General-Use Multimodal AI Platforms for Pell and Gregory Classification of Impacted Mandibular Third Molars

**DOI:** 10.7759/cureus.115713

**Published:** 2026-09-03

**Authors:** John A Fregene, Aditya Tadinada

**Affiliations:** 1 Dentistry, University of Connecticut Health, Farmington, USA; 2 Oral and Maxillofacial Radiology, University of Connecticut Health, Farmington, USA

**Keywords:** artificial intelligence, chatgpt, deep learning, dental imaging, grok, impacted third molars, inter-rater agreement, oral surgery, panoramic radiography, pell and gregory

## Abstract

Purpose: The purpose of this study was to evaluate the performance of two general-use artificial intelligence models, ChatGPT and Grok, in classifying impacted mandibular third molars using the Pell and Gregory system on panoramic radiographs, compared with a resident consensus reference standard.

Materials and methods: One hundred panoramic radiographic images of impacted mandibular third molars were independently classified by two blinded resident reviewers using the Pell and Gregory classification system. Resident consensus was defined as exact agreement between both reviewers; the 94 concordant classifications were confirmed by a board-certified oral and maxillofacial radiologist (the second author), blinded to the AI outputs, and served as the reference standard. Cases without consensus were excluded from the AI accuracy analysis. Each image was then submitted individually to ChatGPT-5.2 and Grok 4.1 through their paid-tier chat interfaces, with each case initiated as a new conversation session. Inter-rater reliability was assessed using percent agreement and Cohen's kappa. AI accuracy was calculated as the proportion of correct classifications among consensus cases, and the two models were compared using McNemar's test with continuity correction.

Results: The two resident reviewers reached consensus on 94 of 100 cases, with an observed agreement of 94.0% and a Cohen's kappa of 0.925 (95% CI: 0.867-0.983). ChatGPT correctly classified 19 of 94 consensus cases (20.2%; 95% CI: 12.1-28.3%), while Grok correctly classified 17 of 94 cases (18.1%; 95% CI: 10.3-25.9%). Both models performed only marginally above the 11.1% accuracy expected from random chance across nine classification categories. McNemar's test showed no statistically significant difference between the two models (χ²(1) = 0.045, p = 0.832).

Conclusions: Both ChatGPT and Grok demonstrated poor accuracy in the Pell and Gregory classification of impacted mandibular third molars when compared with resident consensus. These findings suggest that currently available general-use AI models are not reliable for the independent radiographic classification of impacted mandibular third molars and should not replace expert clinical judgment for this task.

## Introduction

Impacted mandibular third molars are among the most commonly evaluated findings in oral and maxillofacial surgery, and their radiographic assessment carries real clinical weight. The position of an impacted third molar on panoramic imaging can influence surgical planning, perceived extraction difficulty, risk to adjacent structures, and referral or treatment decisions. Because of this, structured classification systems such as the Pell and Gregory classification remain widely used for describing the relationship of the impacted tooth to the anterior border of the ramus and the occlusal plane [1]. Even though these systems are well established, their application still depends on human interpretation, which introduces the possibility of inconsistency between evaluators and limits efficiency in high-volume settings [1,2].

Artificial intelligence has increasingly been explored to improve radiographic interpretation in dentistry, including the detection and classification of impacted mandibular third molars. Prior literature suggests that this potential is real, especially when AI systems are specifically designed for dental imaging tasks. A recent review of deep learning on panoramic radiographs reported that published models achieved diagnostic accuracies ranging from 78.91% to 90.23% for identifying impacted mandibular third molars, while studies evaluating the relationship between impacted third molars and the mandibular canal reported accuracies ranging from 72.32% to 99.00% [3]. More recent work has gone beyond simple detection and has attempted automated classification using established oral surgery frameworks such as the Pell and Gregory and Winter classifications, suggesting that AI may eventually have practical value as a decision-support tool in third molar assessment [4,5].

At the same time, an important distinction exists between purpose-built deep learning systems and the general-use multimodal AI engines that are now available to the public. Most of the stronger performance reported in the literature has come from models trained on curated radiographic datasets for narrow image-analysis tasks [1,2]. That is not the same as asking an everyday AI platform to interpret a panoramic radiograph in a real-world workflow. This distinction matters because if general-use AI engines are ever going to become clinically useful in oral surgery practice, they must demonstrate reliable performance on focused imaging tasks under conditions that resemble actual use rather than optimized development settings.

Current evidence suggests that this gap remains substantial. In a 2025 study evaluating ChatGPT-4o on orthopantomograms of lower third molars, the model achieved an accuracy of only 38.44%, and the authors noted frequent partially correct or fabricated interpretations, reinforcing the need for expert review [6]. More broadly, a 2025 systematic review of large language models in dentomaxillofacial radiology concluded that although these systems show promise across diagnostic and educational applications, their current accuracy, completeness, and consistency remain variable and require further validation before routine clinical integration can be justified [7]. Taken together, these findings suggest that while AI research in dental imaging is progressing quickly, the performance of accessible day-to-day AI engines may still fall well below the standard needed for dependable clinical support.

Impacted mandibular third molars provide a useful and clinically relevant test case for this question. They are common, routinely encountered on panoramic imaging, and typically assessed using a structured classification system already familiar to oral surgeons and trainees. As a result, performance on this task may serve as an early indicator of how close general AI engines are to becoming useful adjuncts in oral surgery practice. If these systems can reliably classify impacted molars, that would support the idea that broader clinical applications may be approaching. If they cannot, that limitation is equally important, because it helps define the current boundary between research promise and practical implementation. In that sense, evaluating general-use AI on impacted molar classification may also offer insight into how close the field is to using similar systems for future detection of other oral and maxillofacial pathologies on routine imaging.

The present study was designed to compare two widely available general-use AI models, ChatGPT and Grok, against resident consensus for the Pell and Gregory classification of impacted mandibular third molars on panoramic radiographs. It was hypothesized that both models would classify these impactions substantially below the level of agreement achieved by trained human reviewers.

## Materials and methods

Study design 

This study was a retrospective image-based classification analysis of 100 panoramic radiographic images of impacted mandibular third molars. Each image was independently evaluated by two resident reviewers using the Pell and Gregory classification system [1].

Resident consensus was defined as exact agreement between Resident 1 and Resident 2. The two residents reached consensus on 94 of the 100 cases. These 94 concordant classifications were independently reviewed and confirmed by the second author (A.T.), a board-certified oral and maxillofacial radiologist who was blinded to the AI outputs, and this confirmed set served as the reference standard. The six cases without resident consensus were excluded from the AI accuracy analysis so that the AI outputs were compared only against a verified reference standard.

Image selection and classification procedure 

The dataset consisted of 100 panoramic radiographic images identified through internet searches for impacted mandibular third molars. The search query used was “radiographic assessment of an impacted mandibular third molar (lower wisdom tooth) using the Pell and Gregory classification.” Images were selected based on radiographic clarity so that the impacted mandibular third molar and its relationship to the ramus and occlusal plane could be visually assessed using the Pell and Gregory classification system [1].

All images were selected by the first author (J.A.F.). Candidate images were retrieved through internet searches for impacted mandibular third molars and were reviewed in the order in which they were returned; an image was included if the impacted mandibular third molar was clearly visible and its relationship to the anterior border of the ramus and the occlusal plane could be assessed. Duplicate images were screened for and removed by visual inspection. Images containing arrows, text labels, or overlaid classification markings were excluded. To standardize presentation, each included image was cropped to approximately the same size prior to submission.

This study was determined to be exempt from Institutional Review Board (IRB) oversight. All images were obtained exclusively from publicly available internet sources and contained no patient-identifiable information, including no names, dates of birth, medical record numbers, or facial photographs. No protected health information, patient records, or institutional clinical data were accessed at any point during the study. As such, this study did not constitute human subjects research as defined by federal regulations and did not require formal IRB review or approval.

Both resident reviewers classified all images independently. Each reviewer was blinded to the other reviewer’s scores, and both reviewers were also blinded to the AI classifications at the time of evaluation. After independent review, cases with matching resident classifications were designated as consensus cases and used as the reference standard. Cases with discordant resident classifications were designated as no-consensus cases and excluded from the subsequent AI accuracy analysis.

AI model evaluation 

The study was initiated on March 1, 2026. The latest available versions of the models at the time of analysis were used, specifically ChatGPT-5.2 (OpenAI, accessed March 2026) and Grok 4.1 (accessed March 2026). Each panoramic radiographic image was evaluated using the same standardized prompt, which was developed from the original Pell and Gregory classification criteria. The prompt provided explicit definitions for the tooth's relationship to the anterior border of the mandibular ramus (Classes I-III) and its depth relative to the occlusal plane and cervical line of the adjacent mandibular second molar (Positions A-C). The model was instructed to combine these two components and to return only one of nine possible classification labels: 1A, 1B, 1C, 2A, 2B, 2C, 3A, 3B, or 3C. The 1933 Pell and Gregory article was uploaded with each image as an additional reference to provide the models with the original classification criteria.

Each image was submitted individually in a new conversation session using identical prompt wording to prevent contextual carryover between cases; no follow-up questions, prompt modifications, corrections, or additional clinical information were provided. Microsoft PowerPoint was used solely as an organizational and transfer tool to download and arrange the images prior to upload, with no enhancement, filtering, or adjustment of contrast, brightness, or resolution. No copyrighted material was reproduced, redistributed, or republished through this process; all images were obtained from publicly available internet sources and were used exclusively for non-commercial, internal research analysis consistent with fair-use principles, and no source images are reproduced within this manuscript. Both platforms were used at their default settings through the paid-tier chat interface, and the same prompt and configuration were applied to every case throughout testing. Model outputs were recorded and compared against the resident consensus classification, and AI performance was evaluated in the consensus cases.

The full standardized prompt submitted with each image was as follows:

You are assisting with a radiographic classification task. The attached image is a panoramic radiograph showing an impacted mandibular third molar. Classify this tooth using the Pell and Gregory classification system, applying the criteria in the attached 1933 Pell and Gregory reference.

Assign the classification in two independent dimensions:

Relationship of the impacted tooth to the anterior border of the mandibular ramus (ramus relationship):

Class I: There is sufficient space between the anterior border of the ramus and the distal aspect of the second molar to accommodate the mesiodistal crown width of the third molar.

Class II: The space between the anterior border of the ramus and the distal aspect of the second molar is less than the mesiodistal crown width of the third molar.

Class III: All or most of the third molar is located within the ramus.

Depth of the impacted tooth relative to the occlusal plane and the cervical line of the adjacent mandibular second molar (depth/position):

Position A: The highest portion of the impacted tooth is at or above the occlusal plane of the second molar.

Position B: The highest portion of the impacted tooth is below the occlusal plane but at or above the cervical line of the second molar.

Position C: The highest portion of the impacted tooth is below the cervical line of the second molar.

Combine the two components and return exactly one of the following nine labels, and nothing else: 1A, 1B, 1C, 2A, 2B, 2C, 3A, 3B, 3C. Provide only the single final classification label. Do not provide explanations, alternatives, ranges, or any additional text.

Statistical analysis 

Descriptive statistics were used to summarize the total number of images, the number and proportion of consensus and no-consensus cases, and the distribution of Pell and Gregory classes among consensus cases. Categorical variables were reported as counts and percentages.

Inter-rater reliability between the two resident reviewers was assessed using percent agreement and Cohen's kappa [8]. Percent agreement was calculated as the proportion of cases in which the two residents assigned the same Pell and Gregory classification out of the total 100 images. Cohen's kappa was used to assess agreement beyond chance, calculated as

$&kappa; = \frac{(P_{o} &minus; P_{e})}{(1 &minus; P_{e})}$

where P_o_ represents observed agreement and P_e_ represents chance-expected agreement derived from the marginal distributions of each reviewer across all nine Pell and Gregory categories. Unweighted kappa was selected because the Pell and Gregory system combines two independent classification dimensions (the relationship to the ramus (Class 1, 2, or 3) and the depth relative to the occlusal plane (Position A, B, or C)) into a single composite label. Because these two dimensions are independent and do not form a single ordinal scale, applying a linear or quadratic weighting scheme to the combined nine-category label would not be clinically meaningful. A 95% confidence interval for kappa was calculated using the standard error approximation [9]:

$SE(&kappa;) = \sqrt{\frac{Pₒ(1 &minus; Pₒ)}{n(1 &minus; Pₑ)&sup2;}}$

For AI evaluation, overall accuracy was calculated separately for ChatGPT and Grok as the proportion of correct classifications among consensus cases only. A 95% confidence interval for each accuracy estimate was calculated using the Wald method. To contextualize AI performance, the expected accuracy under random chance assignment across nine equally distributed categories (1/9 = 11.1%) was reported as a baseline reference. Because both models were tested on the same set of consensus cases, their performances were compared using McNemar's test [10] with continuity correction [11] for paired binary outcomes. McNemar's test is a non-parametric statistical test designed to compare two classifiers evaluated on the same set of observations. It specifically examines whether the pattern of discordant outcomes (cases where one model was correct and the other was not) differs significantly between models. The McNemar chi-squared statistic was calculated as

$&chi;&sup2; = \frac{(|b &minus; c| &minus; 1)&sup2;}{b + c}$

where b and c represent the discordant cell counts. This test is particularly appropriate when the same images are assessed by two different AI systems, as in the present study. Statistical significance was set at p < 0.05. Statistical analyses were performed using Google Sheets.

## Results

Study sample and inter-rater agreement 

A total of 100 panoramic radiographic images of impacted mandibular third molars were included in the study. The two resident reviewers reached exact agreement on 94 of 100 cases (94.0%) and disagreed on 6 cases (6.0%), yielding an overall observed agreement (P_o_) of 94.0%. The chance-expected agreement (P_e_), calculated from the marginal distributions of each reviewer across all nine Pell and Gregory categories, was 20.4%. Applying Cohen's kappa formula, κ = (0.94 − 0.204) / (1 − 0.204), the inter-rater kappa was 0.925 (95% CI: 0.867-0.983), indicating excellent agreement according to the Landis and Koch classification [12]. The two largest contributors to chance-expected agreement were the 2B category (9.00%) and the 2A category (6.76%), reflecting the high frequency of these classifications in both reviewers' marginal distributions. These values are derived probabilities from the marginal distributions and therefore have no corresponding counts.

Because resident consensus served as the reference standard for AI evaluation, the six no-consensus cases were excluded from model accuracy calculations. As a result, ChatGPT and Grok were each evaluated on 94 consensus cases (Table 1).

**Table 1:** Study sample and inter-rater agreement (N = 100 panoramic images) Inter-rater reliability between the two resident reviewers was assessed by percent agreement and unweighted Cohen's kappa; the 95% CI for kappa was derived from the large-sample standard error approximation. Chance-expected agreement (P_e_) is a derived probability from the reviewers' marginal distributions and therefore has no corresponding count.

Variable	Value
Total panoramic images reviewed	100
Consensus cases	94 (94.0%)
No-consensus cases	6 (6.0%)
Observed agreement (P_o_)	94% (94/100)
Chance-expected agreement (P_e_)	20.4%
Cohen’s kappa	0.925 (95% CI: 0.867-0.983)

Distribution of Pell and Gregory classifications 

**Figure 1:**
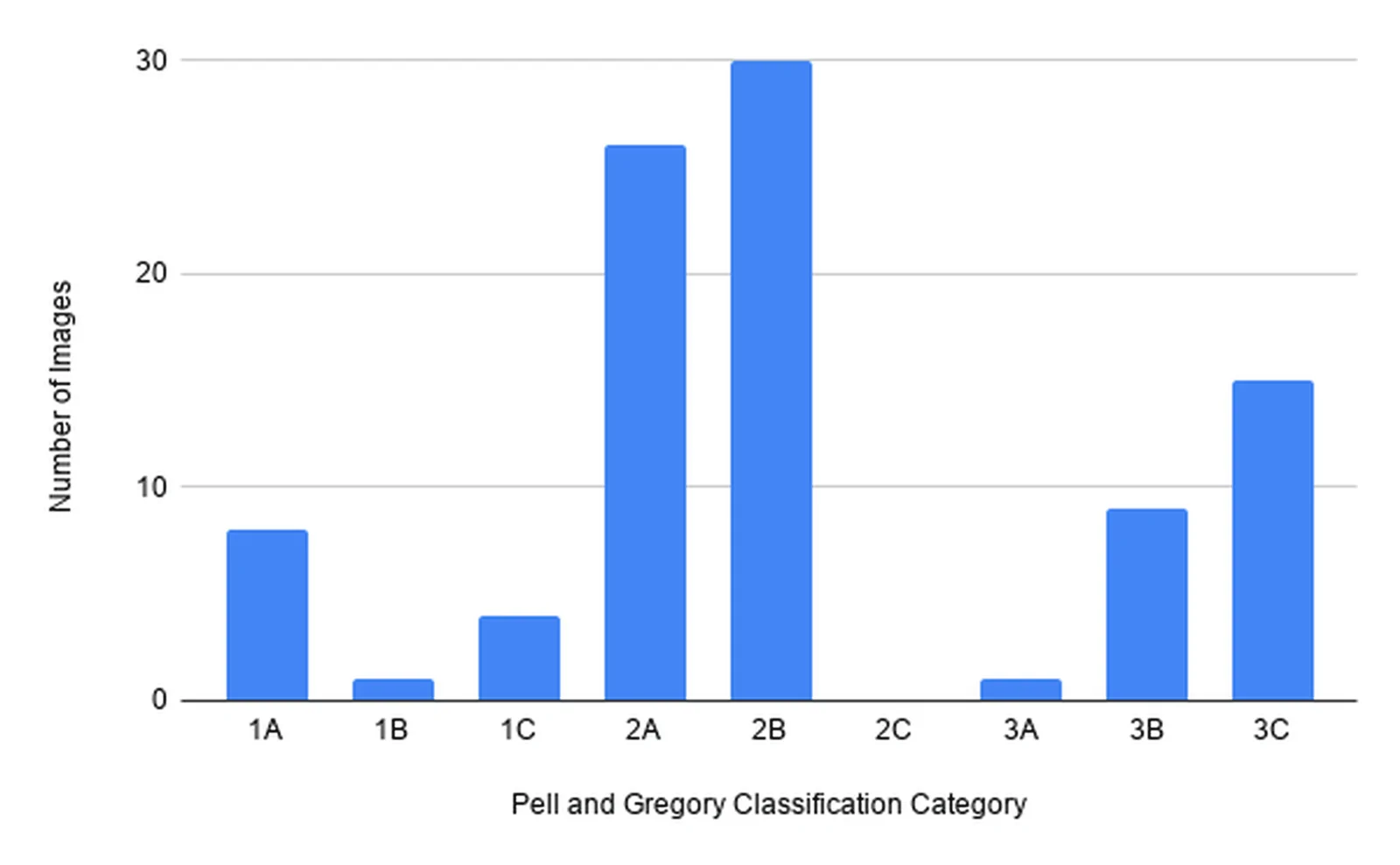
Distribution of Pell and Gregory classifications among consensus cases (n = 94) Bars represent the number of panoramic radiographic images assigned to each Pell and Gregory category by resident consensus: 1A, 8 (8.5%); 1B, 1 (1.1%); 1C, 4 (4.3%); 2A, 26 (27.7%); 2B, 30 (31.9%); 2C, 0 (0.0%); 3A, 1 (1.1%); 3B, 9 (9.6%); and 3C, 15 (16.0%). Percentages are based on the 94 consensus cases. No case was classified as 2C. The six no-consensus cases were excluded. Descriptive data only; no statistical test was applied.

Among the 94 consensus cases, the most common Pell and Gregory classifications were 2B (30/94, 31.9%) and 2A (26/94, 27.7%), followed by 3C (15/94, 16.0%) and 3B (9/94, 9.6%). Less common classifications included 1A (8/94, 8.5%), 1C (4/94, 4.3%), 1B (1/94, 1.1%), and 3A (1/94, 1.1%); no cases were assigned to category 2C (0/94, 0%). The 2A and 2B categories together accounted for 56 of 94 consensus cases (59.6%), while the remaining categories were sparsely represented (Figure 1 and Table 2). 

**Table 2:** Distribution of Pell and Gregory classifications among consensus cases (n = 94) Values are counts (n) and percentages of the 94 consensus cases. All nine Pell and Gregory categories are shown, including category 2C, to which no case was assigned (0/94, 0.0%). Percentages are rounded to one decimal place and may not sum to exactly 100% because of rounding. No inferential testing was performed.

Pell and Gregory classification	n	Percentage of consensus cases
1A	8	8.50%
1B	1	1.10%
1C	4	4.30%
2A	26	27.70%
2B	30	31.90%
2C	0	0%
3A	1	1.10%
3B	9	9.60%
3C	15	16%
Total	94	100%

AI model performance 

**Figure 2:**
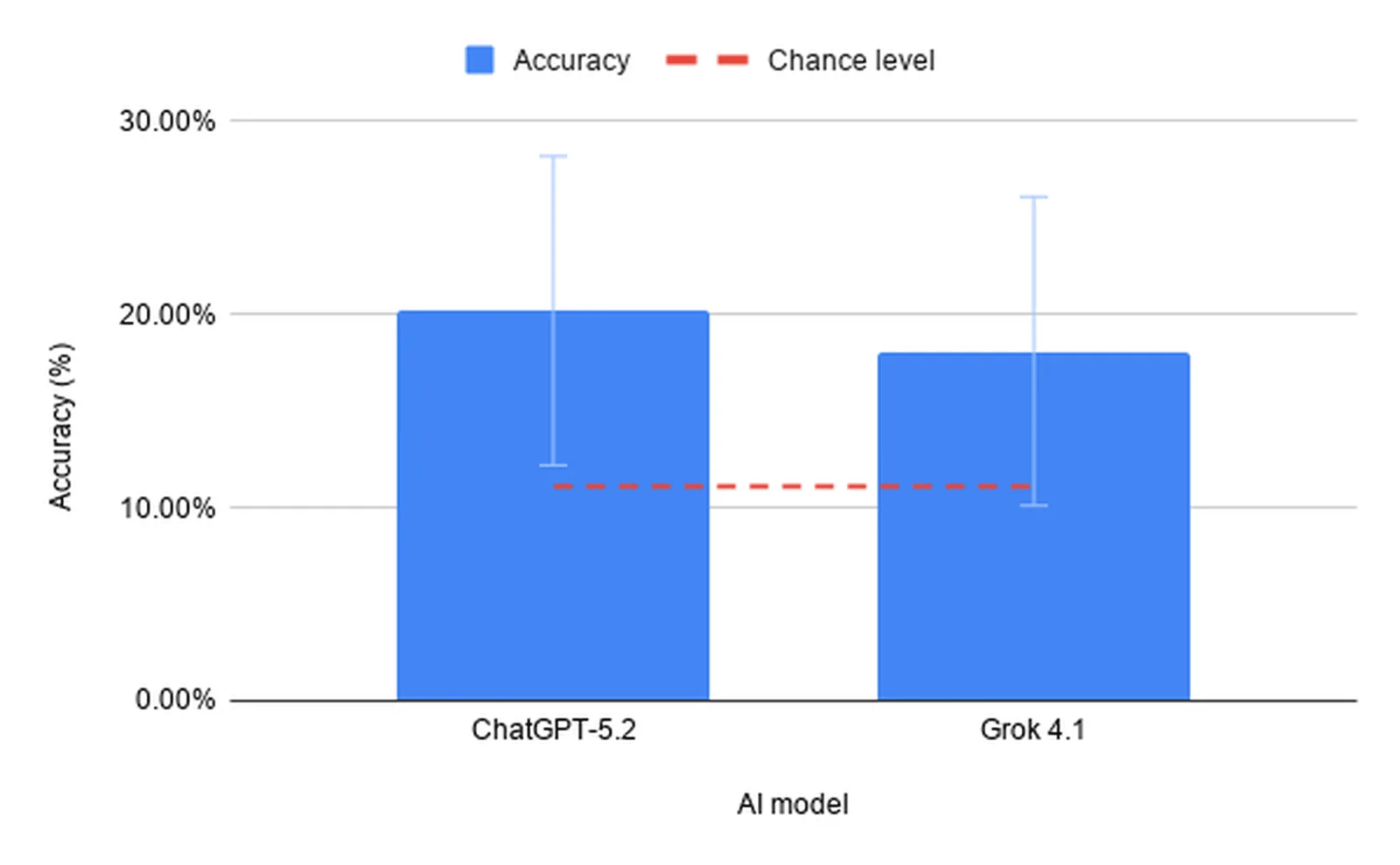
Accuracy of ChatGPT and Grok compared with resident consensus (n = 94) Bars represent overall classification accuracy, defined as the proportion of the 94 consensus cases correctly classified: ChatGPT-5.2, 19/94 (20.2%); Grok 4.1, 17/94 (18.1%). Error bars denote 95% confidence intervals calculated using the Wald method (ChatGPT-5.2, 12.1–28.3%; Grok 4.1, 10.3–25.9%). The red dashed line indicates the 11.1% accuracy expected from random assignment across the nine Pell and Gregory categories.

Using resident consensus as the reference standard, ChatGPT correctly classified 19 of 94 consensus cases, corresponding to an overall accuracy of 20.2% (95% CI: 12.1-28.3%). Grok correctly classified 17 of 94 consensus cases, corresponding to an overall accuracy of 18.1% (95% CI: 10.3-25.9%). For context, random assignment across nine equally distributed Pell and Gregory categories would yield an expected accuracy of 11.1% (1/9), indicating that both models performed only marginally above the chance level (Figure 2 and Table 3). 

**Table 3:** Accuracy of ChatGPT and Grok using resident consensus as the reference standard (n = 94) Values are counts and accuracy (%), with 95% confidence intervals calculated using the Wald method. The paired between-model comparison is reported in Table 4.

Model	Correct, n (%)	Incorrect, n (%)	Total consensus cases, n	Accuracy, % (95% CI)
ChatGPT	19 (20.2)	75 (79.8)	94	20.2% (95% CI: 12.1-28.3)
Grok	17 (18.1)	77 (81.9)	94	18.1% (95% CI: 10.3-25.9)

Paired comparison between ChatGPT and Grok 

Because both models were evaluated on the same 94 consensus cases, their performance was directly compared using McNemar's test with continuity correction. Among the 94 cases, 7 were correctly classified by both models, 65 were incorrectly classified by both models, 12 were correctly classified by ChatGPT only, and 10 were correctly classified by Grok only. The McNemar chi-squared statistic, calculated from the discordant cells as χ² = (|12 − 10| − 1)² / (12 + 10), was χ²(1) = 0.045, p = 0.832, indicating no statistically significant difference between the two models. The 95% confidence intervals for the two models were wide and overlapping (ChatGPT-5.2, 12.1-28.3%; Grok 4.1, 10.3-25.9%), indicating that the observed 2.1-percentage-point difference in accuracy was within the range of sampling variability (Table 4).

**Table 4:** Paired comparison of ChatGPT and Grok classifications on consensus cases Paired comparison of ChatGPT-5.2 and Grok 4.1 classifications on consensus cases (n = 94). Values in the 2 × 2 matrix are counts (n) with percentages of the 94 consensus cases; cells represent concordance and discordance between the two models relative to the resident consensus reference standard. The final row reports the between-model comparison: discordant pairs (b = 12 correct by ChatGPT only; c = 10 correct by Grok only) were compared using McNemar's test with continuity correction, χ²(1) = 0.045, p = 0.832. df = degrees of freedom. Statistical significance was set at p < 0.05.

ChatGPT	Grok correct, n (%)	Grok incorrect, n (%)	Total, n (%)
ChatGPT correct	7 (7.4)	12 (12.8)	19 (20.2)
ChatGPT incorrect	10 (10.6)	65 (69.1)	75 (79.8)
Total	17 (18.1)	77 (81.9)	94 (100)
McNemar's test with continuity correction	χ²(1) = 0.045	p = 0.832	

## Discussion

Main findings 

This study compared two general-use AI models, ChatGPT and Grok, with resident consensus for Pell and Gregory classification of impacted mandibular third molars [1]. The two residents applied the classification with almost perfect agreement on the Landis and Koch scale, establishing a reliable human reference standard [12]. Against that standard, both models correctly classified only about one-fifth of the consensus cases, marginally above the accuracy expected from random assignment across nine categories, and the two models did not differ significantly. Neither model therefore demonstrated meaningful classification ability, and neither can be considered superior to the other; the results should be interpreted not as one model outperforming the other but as evidence that both general-use engines are currently unreliable for this task. Despite growing interest in AI-assisted dental imaging, currently available general-use models are not reliable for independent Pell and Gregory classification of impacted mandibular third molars.

Importance of resident agreement 

The high inter-rater agreement between the two residents strengthens the interpretation of the AI results, indicating that the Pell and Gregory classification was applied consistently by trained human reviewers [2] and that the observed agreement far exceeded chance rather than reflecting an artifact of category distribution. Because the AI models were evaluated only on consensus cases, their low accuracy is less likely to reflect uncertainty in the reference standard or ambiguity in the dataset. Instead, the findings more likely reflect limitations in the ability of general-use AI models to interpret panoramic radiographs using a structured oral surgery classification system.

Comparison with previous AI literature 

Previous studies have shown that AI can perform well in dental radiographic interpretation when models are purpose-built for imaging tasks. Deep learning models have achieved diagnostic accuracies of 78.91% to 90.23% for identifying impacted mandibular third molars and 72.32% to 99.00% for assessing their relationship to the mandibular canal, and machine learning has been applied to preinterventional third molar assessment [3]. These systems, however, are trained on curated radiographic datasets for narrow tasks. This is different from the present study, in which general-use AI models were asked to classify impacted mandibular third molars in a way that more closely resembles how these tools may be used in a real-world setting. The lower accuracy found in this study suggests that the performance of specialized dental AI models cannot be directly extrapolated to publicly available general-use AI engines such as ChatGPT and Grok [6]. Although AI-assisted dental imaging continues to develop, the current findings show that general-use AI models may still have major limitations when asked to complete a focused oral surgery classification task.

Difference between purpose-built AI and general-use AI 

The findings of this study highlight the important differences between purpose-built dental AI systems and general-use AI models. Purpose-built AI systems are usually trained on specific radiographic images and are designed to complete one narrow task, such as detecting an impacted tooth or identifying an anatomic relationship [5]. In contrast, general-use AI models are designed to answer a wide range of questions and interpret many different types of images, but they may not be specifically trained to apply dental classification systems [13]. Pell and Gregory classification requires the model to evaluate the relationship of the impacted third molar to the anterior border of the ramus and the occlusal plane. This requires detailed radiographic interpretation and a clear understanding of oral surgery anatomy. The poor performance seen in this study suggests that general-use AI models may not yet be able to consistently apply this type of structured classification system, even when the task is familiar to trained dental residents [13].

Possible reasons for poor AI performance 

There are several possible reasons why both AI models performed poorly in this study. Pell and Gregory classification is based not only on recognizing the impacted tooth but also on correctly judging the tooth’s position in relation to specific anatomic landmarks. Panoramic radiographs can also vary in angulation, contrast, image quality, and anatomic overlap, which may make classification more difficult. Even for humans, some cases can be more difficult to classify when the tooth position is close to the border between two categories. However, because the AI models were evaluated only on resident consensus cases, poor performance is less likely to be due to unclear images alone. Instead, the findings suggest that the models may have difficulty identifying the exact radiographic landmarks needed for Pell and Gregory classification. General-use AI models may also provide a definitive answer even when uncertain, which can make them appear more confident than warranted in a clinical setting [6,13].

Clinical relevance 

The clinical relevance of these findings is considerable because impacted mandibular third molars are commonly evaluated in oral and maxillofacial surgery. Pell and Gregory classification can help describe the position of the tooth, communicate surgical difficulty, and support treatment planning [1]. If an AI model gives an incorrect classification, it could affect how a case is interpreted, especially by students or clinicians who may rely too heavily on the AI output. While AI may eventually become useful as a support tool, the findings of this study suggest that general-use AI models should not be used independently for this type of radiographic classification. Expert review is still needed, and AI-generated classifications should be interpreted with caution [6]. At this stage, these models may have more value as educational or exploratory tools rather than as dependable clinical decision-making tools.

Limitations 

Dataset Size 

One limitation of this study is the relatively small dataset size. A total of 100 panoramic radiographic images were reviewed, with 94 consensus cases included in the AI accuracy analysis. Although this sample size allowed for an initial comparison between resident consensus and AI model performance, a larger dataset would help make the findings more generalizable. A larger number of cases would also allow for better evaluation across each Pell and Gregory classification category. Since some categories were represented by only a small number of cases, future studies with larger datasets would be needed to better understand how these AI models perform across all classification types.

Uneven Distribution of Pell and Gregory Classifications

Another limitation is that the Pell and Gregory classifications were not evenly distributed across the dataset. The consensus cases were mostly made up of 2A and 2B classifications, while some categories had very few examples. This uneven distribution limits the ability to determine whether the AI models performed better or worse on specific Pell and Gregory categories. For example, if a category only had one case, it is not possible to make a strong conclusion about model performance for that classification. Future studies should try to include a more balanced distribution of Pell and Gregory classifications so that accuracy can be compared more clearly across each category.

Use of Publicly Available Images

This study used panoramic radiographic images that were obtained from publicly available internet sources. This allowed the study to be completed without patient-identifiable information or protected health information. However, this also creates a limitation because publicly available images may not fully represent the type of radiographs seen in a true clinical setting. Public images may vary in quality, angulation, labeling, and image selection. Some images may also be more educational or obvious examples than consecutive clinical cases. Because of this, the findings may not fully reflect how these AI models would perform on a larger clinical dataset from an oral surgery or radiology department. In addition, no prespecified search or screening protocol, systematic sampling method, formal duplicate or near-duplicate screening, per-image source and licensing documentation, or predefined inclusion/exclusion criteria were applied during image selection, and no supplementary curation table is provided. Images were selected pragmatically on the basis of radiographic clarity from publicly available internet sources. These constraints limit the reproducibility of the sampling process and should be considered when interpreting the generalizability of the results.

Image Selection and Curation

No prespecified image search, screening, or curation protocol was applied. Images were identified through general internet searches without a systematic sampling method, and the study did not perform formal duplicate or near-duplicate screening, did not document the source, access date, or licensing status of each image, did not apply predefined inclusion and exclusion criteria for annotations or required anatomical landmarks, and did not verify image provenance. As a result, per-image source documentation is not available. Future studies should apply a prespecified search and screening protocol, excluding duplicate or near-duplicate images, annotated images, images lacking the required landmarks, and images of uncertain provenance, and should report the source, access date, licensing status, eligibility assessment, and preprocessing for every included image (e.g., in a supplementary table).

Possible Training-Data Overlap

Because the images were obtained from publicly available internet sources, some may have been present in the training data of ChatGPT or Grok. Although both models performed only marginally above chance, potential overlap between the evaluation images and model training corpora cannot be excluded and could bias accuracy estimates in either direction. This risk is inherent to evaluating general-use models on public images and reinforces the need for future studies using curated, non-public clinical datasets with documented provenance.

Model Version Changes

Another limitation is that general-use AI models are frequently updated. This study evaluated the model versions available at the time of analysis, but future versions of ChatGPT or Grok may perform differently. Because these platforms can change over time, results from one version may not fully apply to later versions. This makes research on general-use AI time-sensitive. Future studies should clearly report the model version, date of access, and any relevant settings used during testing so that results can be better interpreted and compared across studies.

No Evaluation of Partial Correctness

This study evaluated exact final classification accuracy only. This means that an AI response was counted as correct only if it matched the full resident consensus Pell and Gregory classification. However, Pell and Gregory classification has two parts: the relationship to the ramus and the depth of the tooth relative to the occlusal plane. It is possible that a model may correctly identify one part of the classification but miss the other part. This study did not separately evaluate partial correctness. Future studies should consider separating the classification into its individual components to better understand whether AI models struggle more with ramus relationships, depth classification, or both.

No Assessment of Model Repeatability

A further limitation is that model repeatability was not evaluated. Each panoramic image was submitted to each model only once, as a single new conversation session, so the study assessed classification accuracy against the resident-consensus reference standard rather than the consistency of model outputs across repeated submissions. Because general-use AI models can produce non-deterministic outputs, the same image submitted multiple times may yield different Pell and Gregory classifications. Consequently, the output consistency, or intra-model repeatability, of ChatGPT and Grok for this task remains unknown and cannot be inferred from the present accuracy data.

Future directions 

Larger Clinical Datasets

Future studies should evaluate general-use AI models using larger clinical datasets. A larger number of panoramic radiographs would allow for stronger conclusions and better evaluation across all Pell and Gregory categories. Ideally, future datasets should include images from actual clinical settings and should include a more balanced number of cases in each classification group. Multi-institutional datasets may also be useful because they would include more variation in image quality, patient anatomy, and radiographic technique. This would help determine whether the poor AI performance seen in this study remains consistent across broader clinical samples.

Comparison With Dental-Specific AI Systems

Future research should also compare general-use AI models with dental-specific or oral radiology-specific AI systems. The present study focused on ChatGPT and Grok, which are general-use models and are not specifically designed for Pell and Gregory classification. Comparing these models with purpose-built dental AI systems would help show whether the low accuracy is mainly due to the type of model being used or the difficulty of the classification task itself [5,14]. This type of comparison would also help determine whether general-use AI can realistically be used in oral surgery imaging or whether dental-specific models are needed for dependable performance.

Different Prompting Strategies

Future studies should evaluate whether different prompting strategies improve AI performance. In this study, the models were asked to provide a single final Pell and Gregory classification. However, future prompts could be more structured by asking the model to first identify the anterior border of the ramus, then evaluate the occlusal plane, and then provide the final classification. Other prompts could include written classification criteria, reference diagrams, or step-by-step instructions. Studying different prompt formats would help determine whether poor performance is due to the model’s image interpretation ability or whether clearer prompting can improve classification accuracy.

Assessment of Model Repeatability

Future studies should also evaluate the repeatability of general-use AI models by submitting each image multiple times, both within a single session and across separate sessions. Because these models can generate non-deterministic outputs, repeated submissions of identical images would allow calculation of intra-model agreement and would clarify whether classification errors reflect stable model behavior or session-to-session variability. Reporting both accuracy and repeatability would provide a more complete picture of whether general-use AI engines can be depended upon for structured oral surgery classification tasks.

Educational Use

Although the AI models were not reliable for independent classification in this study, future research should evaluate whether they may still have value as educational tools. General-use AI models may be useful for explaining the Pell and Gregory system, helping students review classification criteria, or providing practice cases with supervision. However, because the models showed low accuracy, their responses should not be used without expert review [7]. In an educational setting, AI may be most useful when it is used as a supportive tool rather than as the final source of classification. Further studies are needed to determine whether these models can help students learn oral surgery classification systems while still maintaining accuracy and patient safety.

## Conclusions

In this convenience sample of publicly accessible panoramic images with concordant resident classifications, both ChatGPT and Grok demonstrated low single-run exact-match accuracy for Pell and Gregory classification of impacted mandibular third molars, and no statistically significant difference was detected between them. Because the reference categories were markedly imbalanced, these accuracies fell below dataset-informed benchmarks (e.g., an always-2B classifier, ~31.9%; a prevalence-matched random classifier, ~22.2%), so accuracy was not meaningfully above an appropriate chance baseline. These findings should not be generalized to clinical populations until confirmed using systematically sampled clinical images, an expert-adjudicated reference standard, archived prompts, and repeated model evaluations.

However, the low performance of these models should not be viewed only as a limitation but also as an opportunity for future improvement. As dental-specific AI models continue to advance, general-use AI systems will need to become more accurate, consistent, and clinically reliable if they are going to compete with models specifically designed for dental imaging. Future development should focus on improving the ability of general-use AI engines to recognize oral and maxillofacial anatomy, apply structured classification systems, and provide dependable support for clinical interpretation.

Ultimately, this study helps define the current boundary between AI potential and clinical readiness. At this stage, general-use AI engines should not be used as independent diagnostic tools for impacted mandibular third molar classification. With further validation, improved training, and stronger image-analysis capabilities, these systems may eventually become useful as adjuncts for dental students, residents, and clinicians. The future goal should be to use AI as a tool that supports diagnosis, strengthens education, and improves clinical efficiency while maintaining expert human oversight.
